# Are Pharmaceuticals with Evolutionary Conserved Molecular Drug Targets More Potent to Cause Toxic Effects in Non-Target Organisms?

**DOI:** 10.1371/journal.pone.0105028

**Published:** 2014-08-20

**Authors:** Sara Furuhagen, Anne Fuchs, Elin Lundström Belleza, Magnus Breitholtz, Elena Gorokhova

**Affiliations:** Department of Applied Environmental Science, Stockholm University, Stockholm, Sweden; Florida International University, United States of America

## Abstract

The ubiquitous use of pharmaceuticals has resulted in a continuous discharge into wastewater and pharmaceuticals and their metabolites are found in the environment. Due to their design towards specific drug targets, pharmaceuticals may be therapeutically active already at low environmental concentrations. Several human drug targets are evolutionary conserved in aquatic organisms, raising concerns about effects of these pharmaceuticals in non-target organisms. In this study, we hypothesized that the toxicity of a pharmaceutical towards a non-target invertebrate depends on the presence of the human drug target orthologs in this species. This was tested by assessing toxicity of pharmaceuticals with (miconazole and promethazine) and without (levonorgestrel) identified drug target orthologs in the cladoceran *Daphnia magna*. The toxicity was evaluated using general toxicity endpoints at individual (immobility, reproduction and development), biochemical (RNA and DNA content) and molecular (gene expression) levels. The results provide evidence for higher toxicity of miconazole and promethazine, i.e. the drugs with identified drug target orthologs. At the individual level, miconazole had the lowest effect concentrations for immobility and reproduction (0.3 and 0.022 mg L^−1^, respectively) followed by promethazine (1.6 and 0.18 mg L^−1^, respectively). At the biochemical level, individual RNA content was affected by miconazole and promethazine already at 0.0023 and 0.059 mg L^−1^, respectively. At the molecular level, gene expression for cuticle protein was significantly suppressed by exposure to both miconazole and promethazine; moreover, daphnids exposed to miconazole had significantly lower vitellogenin expression. Levonorgestrel did not have any effects on any endpoints in the concentrations tested. These results highlight the importance of considering drug target conservation in environmental risk assessments of pharmaceuticals.

## Introduction

Pharmaceutical residues are ubiquitous in the environment due to extensive human and veterinary use. Sewage treatment plants cannot successfully eliminate all pharmaceuticals that remain in the effluent either in their original form or as metabolites with residual activities [1]. To achieve optimal therapeutic function, pharmaceuticals are chemically designed to fit specific molecular targets [2], [3], which are often evolutionary conserved and have orthologs in a variety of organisms [4]. This raises concerns regarding potential impacts of pharmaceutical pollution on non-target organisms that have high similarity of molecular targets, such as receptors and enzymes, with humans (so called, the read-across hypothesis [5]). Adverse effects on these organisms may occur already at environmentally relevant concentrations that are well below LC_50_ (i.e., concentration at which 50% mortality occurs) determined in acute tests. The synthetic estrogen 17α-ethinyl estradiol (EE2) is a high-profile case when acute assays were unable to provide sufficient toxicological information [6]. The EE2 enters the environment through sewage treatment plants and at concentrations in the ng L^−1^ range causes feminization in fish, where estrogen receptors are present [7]–[9]. However, the LC_50_ values obtained in the acute mortality tests on fish are in the range of mg L^−1^ [10]. In contrast to fish, no receptors for EE2 have been found in crustaceans, in which the observed effects of EE2 are minor [11], [12]. Compared to vertebrates, invertebrates have fewer identified orthologs to human drug targets. However, many biochemical and physiological systems are evolutionarily conserved among mammals and invertebrate non-target species, including crustaceans [4]. For instance, the pharmacological target of serotonin reuptake inhibitors is conserved in invertebrates and have consequences for the toxicity of this group of pharmaceuticals [13]. Therefore, knowledge of drug target conservation status could assist in study design, selection of appropriate test species and endpoints as well as data interpretation; this approach is currently referred to as “intelligent testing” in environmental risk assessment [4], [14]. According to the read-across hypothesis, a pharmacological effect in non-target species will occur if the drug target is conserved and the plasma concentration of a drug is similar to the human therapeutic concentrations [5]. However, detecting pharmacological response before a toxicological one is challenging, particularly in small invertebrates, in which drug target orthologs may be involved in other metabolic pathways than their counterparts in vertebrates and the effective dose may differ [15]. Therefore, testing read-across hypothesis *stricto sensu*, i.e. linking haemolymph (analogous to plasma concentrations in fish) and mode of action based effects [5], is not readily applicable to microcrustaceans used in ecotoxicological testing. Yet, it should be possible to use information on various downstream toxicological effects derived from measuring multiple endpoints across different levels of biological organization for drug screening.

In this study, we hypothesized that the capacity of a pharmaceutical to cause an effect in non-target organisms depends on the presence of human drug target orthologs in the organism. This hypothesis was tested using pharmaceuticals selected to represent those with and without an identified human drug target ortholog in the cladoceran *Daphnia magna*, a common model test species in ecotoxicology. For two of the pharmaceuticals, miconazole and promethazine, an ortholog for the human target calmodulin (CaM) has been identified in *Daphnia*, whereas the third pharmaceutical, levonorgestrel, does not have an identified target ortholog for progesterone or estrogen in this genus [4]. Miconazole belongs to the imidazoles, a family of drugs that deplete ergosterol and thus alter membrane structure in fungi [16], but it has also been suggested that imidazoles act as inhibitors of CaM activity [17]. Although promethazine's therapeutic action is to inactivate the H1-receptor [18], it has also been found to be a CaM antagonist [19]. Levonorgestrel is a second generation synthetic progestogen that regulates female reproductive cycle and is used in human contraceptive drugs [20]; to date, no target ortholog has been reported for this drug in invertebrates [4]. More specifically, we hypothesized that miconazole and promethazine, i.e. pharmaceuticals for which a drug target ortholog is present in the test species, will cause toxic effects at lower concentrations compared to levonorgestrel.

To address this hypothesis, we applied a battery of ecotoxicological endpoints at individual, biochemical and molecular levels. Immobility, reproduction and development were measured as individual endpoints since they represent crucial life events in the crustacean life cycle [21]. Feeding inhibition, a commonly used and sensitive endpoint [22] that could have consequences for energy intake, growth and metabolism, was also assessed [23]. Individual RNA and DNA contents were used as biochemical endpoints, as RNA content is a practical and relatively sensitive tool for assessing changes in bulk protein synthesis rate, metabolic performance and growth [24]. Gene expression of vitellogenin and cuticle protein were used as molecular endpoints since they are relevant as indicators of reproductive and developmental effects [25]. In *Daphnia*, expression of these genes has been used to diagnose endocrine disruption as a result of exposure to various contaminants, including pharmaceuticals [25], [26] and metals [27], but also as a part of phototoxicity and oxidative stress responses [25]. Alterations in gene expression can be a direct response or a downstream result of a toxic exposure [28] and could facilitate identification of toxic mechanisms and affected pathways within the adverse outcome pathway concept [29].

## Material and Methods

### Pharmaceuticals

All pharmaceuticals (98% purity) were purchased from Sigma-Aldrich. They were dissolved in dimethyl sulfoxide (DMSO) for use in the bioassays. DMSO volume in test medium corresponded to 0.1‰ of the total volume and solvent control was used in all tests. Water samples were collected for chemical analysis (Tables A and B in Text S1 and Table S1) and stored at −20°C.

### Test organisms

In all bioassays, the neonates (24-h) of *D. magna* used originated from a single clone (environmental pollution test strain Klon 5, the State office for nature, environment, and customer protection North-Rhine Westfalia, Bonn, Germany; originally from the Federal Environment Agency, Berlin, Germany). The animals were cultured in groups of ∼20 individuals in 3-L beakers with M7 medium (OECD standard 202 and 211), and fed a mixture of the green algae *Pseudokirchneriella subcapitata* and *Scenedesmus subspicatus* three times a week.

### Acute and reproduction test

For all three pharmaceuticals, acute toxicity (OECD 202) [30] and reproduction (21-d, OECD 211) [31] tests were conducted, using glass beakers with 50 mL test volume, at a light:dark cycle of 16∶8 h and a temperature of 20±1°C. Immobility tests (48-h, OECD 202) were conducted for miconazole (0.11–0.56 mg L^−1^), promethazine (0.12–9.4 mg L^−1^) and levonorgestrel (0.11–1.7 mg L^−1^), with 4 replicates (5 neonates each) per concentration, and observations every 24 h. Reproduction tests for miconazole (0.00078–0.064 mg L^−1^), promethazine (0.0062–0.53 mg L^−1^) and levonorgestrel (0.013–1.02 mg L^−1^), with 10 replicates per concentration, were conducted using individual daphnids fed *P. subcapitata* (0.1 mg C d^−1^ during the first week and 0.2 mg C d^−1^ during the following two weeks). Test beakers were checked every 24 h and new neonates were noted and removed. Test medium was changed three times per week.

### Exposure for development, RNA/DNA measurements and gene expression analysis

Due to logistic constraints, exposure of animals for assessment of development rate, gene expression and RNA content was conducted on several occasions. For promethazine, these endpoints were measured in a single experiment, whereas exposure for miconazole and levonorgestrel were repeated twice, first for the RNA measurements and then for the development rate and gene expression analyses. Daphnids were exposed individually in 24-well microplates, with 2 mL test solution per well, at the same light and temperature conditions as in the acute and reproduction tests. The test solution and food (*P. subcapitata*, 0.025 mgC animal^−1^) were renewed every second day. For the development rate assessment, the wells (*n* = 130–150) were examined for shed exoskeletons every 24 h, and the current instar was noted. The mean instar at the check point was used as a proxy of development rate. The experiment was terminated when ≥50% of the animals reached instar 3, this occurred on day 2 for promethazine and on day 3 for miconazole and levonorgestrel. Dissolved oxygen saturation and pH were recorded at the start and at the end of the test (Table S1).

Test concentrations for incubations used for RNA and DNA measurements and gene expression analysis were 0.0023 mg L^−1^ for miconazole and 0.059 mg L^−1^ for promethazine corresponding to a factor nine and three below their respective LOEC (lowest observed effect concentration) obtained in the reproduction tests. As no significant reproduction effects were found for levonorgestrel within the concentration range tested, the test concentration for biochemical and molecular endpoints was set to 1.02 mg L^−1^.

All water samples (except accidentally lost samples from immobility and reproduction tests with levonorgestrel) were sent for chemical analysis (see Text S1 for details on the analytical methods).

### Feeding inhibition test

Feeding inhibition tests (24-h) with promethazine (0.12–1.04 mg L^−1^) and levonorgestrel (0.11–1.7 mg L^−1^) were performed according to Barata et al. [22] with some modifications. Exposure was conducted in glass vessels with green alga *P. subcapitata* as a food source (1.5 µg C mL^−1^) in darkness at 20±1°C. Algae concentration (mg L^−1^) was measured at the start and end with Fluorometer Turner designs model 10-AU-000. Feeding rate (mg C animal^−1^ h^−1^) was calculated according to Allen et al. [32].

### RNA and DNA measurement

Ten daphnids per treatment and control were used for the RNA and DNA content measurements. The body length (BL, mm; defined as the distance between the eye and the base of the tail spine) of these animals was measured and used as a measure of body size. Individual RNA content was used as a proxy for protein synthetic capacity, whereas DNA content was determined to detect treatment effects on the relationship between BL and cell number in the animals. The length-measured daphnids were preserved individually in 30 µL RNAlater and stored at −20°C until analysis. Measurements of RNA and DNA followed the method described by Gorokhova and Kyle [33] using FLUOstar Optima (filters: excitation 485 nm, emission 520 nm) and black flat-bottom microplates.

### Gene expression

Real-Time quantitative PCR was used to measure relative difference in mRNA expression of vitellogenin and cuticle protein 12 between the exposed and control animals; as a reference gene, β-actin was used. At the termination of the experiment, three replicates for each treatment and control with 20–25 animals per replicate were sampled in Eppendorf tubes and immediately frozen at −80°C until the RNA extraction. Total RNA was extracted using the RNeasy Mini kit with on-column DNase treatment (Qiagen, UK), following the manufacturer's instructions. RNA concentrations were determined on a NanoPhotometer (Implen, Germany). Reverse transcription and amplification were conducted using Power SYBR Green RNA-to-CT 1-Step Kit (Applied. Biosystems, Foster, CA, USA) in a 20 µL reaction volume according to the protocol recommended by the manufacturer. The primers for a 77 bp fragment of vitellogenin (GenBank accession number: DY037239; forward: 5′-GCGGACGAGGTTGCAAAG-3′ and reverse: 5′-AGGAGCAGGAAGATGTCGTTCT-3′), a 199 bp fragment for cuticle protein 12 (DW985490; forward: 5′-AGCCAGTGGAACTACG-3′ and reverse: 5′-TCCAGCATCATCAGCG-3′), and a 71 bp fragment for β-actin (AJ292554; forward: 5′-CCACACTGTCCCCATTTATGAAG-3′ and reverse: 5′-CGCGACCAGCCAAATCC-3′) were adopted from Soetaert et al. [26] and Kim et al. [25]. The reaction plate was subjected in StepOne (Applied BioSystems) real time PCR with reverse transcription. The thermal profile consisted of 30 min of reverse transcription at 48°C one cycle and 10 min of polymerase activation at 95°C, followed by 40 cycles of PCR at 95°C for 15 s and 60°C for 60 s. Following amplification, a melting curve analysis was performed to verify the authenticity of the amplified product by its specific melting temperature. Validation experiments confirmed that the efficiencies of the target and endogenous control (β-actin) amplifications were similar (95–98%). The comparative threshold cycle method [34] was used to assess the relative levels of target gene mRNAs normalized to those of β-actin.

### Statistics

In acute tests, LC_50_ values were calculated using Probit (version 2.3). Reproduction effects, i.e. mean number of offspring, and mortality were analyzed with ANOVA and LSD post hoc using SPSS (PASW Statistics 18); Levene's test was used to assess the equality of variances. General linear models were run in R 2.13.2 to evaluate treatment effects on the relationships between RNA content and BL and between DNA content and BL; all data for BL, RNA and DNA content were log transformed. If the interaction between BL and treatment was non-significant (p>0.05), it was removed to test treatment effect on RNA content. To evaluate differences in mRNA expressions and development between the exposed and controls animals, unpaired t-test was applied.

## Results

### Acute and reproduction toxicity

Both promethazine and levonorgestrel were present in the water phase. By contrast, miconazole was not found in the water phase; yet the exposure effects on the immobility and reproduction were evident from the dose-response curve (Figures S1 and S2). Moreover, miconazole was the most toxic among the three pharmaceuticals tested, with the lowest LC_50_ and LOEC values for immobility and reproduction, respectively (Table 1). Effects of promethazine were observed at five- (acute toxicity) and eight-fold (reproductive toxicity) higher concentrations than for miconazole. No effects of levonorgestrel were observed within the range of the concentrations tested. The pH and oxygen levels were all within acceptable limits throughout all exposures.

**Table 1:** Effect concentrations obtained in acute toxicity test (OECD 202) (LC_50_; mg L^−1^), reproduction test (OECD 211) (LOEC reproduction and mortality; mg L^−1^) and feeding inhibition test (LOEC; mg L^−1^), and concentration range (Range, mg L^−1^) for the pharmaceuticals tested.

Pharmaceutical	Acute toxicity		Reproduction			Feeding	
	LC_50_	Range	LOEC offspring production	LOEC mortality	Range	LOEC	Range
Miconazole	0.3	0.11–0.56	0.022	0.064	0.00078–0.064	-	-
Promethazine	1.6	0.12–9.40	0.18	>0.53	0.0062–0.53	1.04	0.12–1.04
Levonorgestrel	>1.7	0.11–1.70	>1.02	>1.02	0.013–1.02	>1.7	0.11–1.70

Feeding inhibition test with miconazole could not be conducted due to high toxic effects on the algae.

### Development

**Figure 1:**
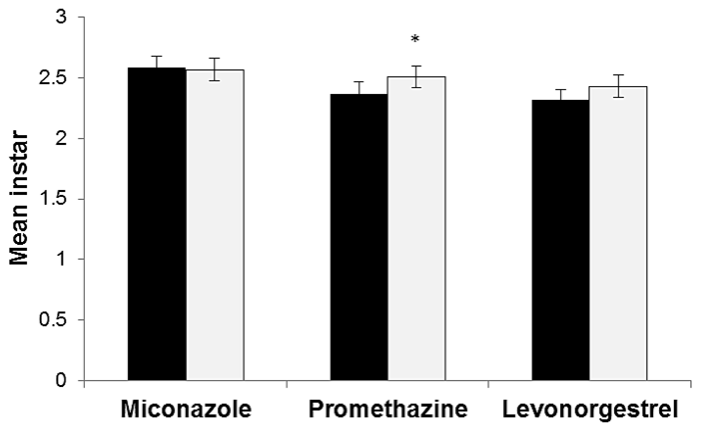
Development and mean instar. Mean instar (with 95% confidence intervals) for the 2-d incubation (n = 10). Test concentrations were 0.0023 mg L^−1^ for miconazole, 0.059 mg L^−1^ for promethazine and 1.02 mg L^−1^ for levonorgestrel. Black bars represent controls and white are the treatments. Asterisk indicates significant level: p≤0.05 (*) determined by an unpaired t-test.

Daphnids exposed to promethazine developed significantly faster than the control animals (t = −2.14, p = 0.034; Figure 1). In the 2-d incubations, no significant effects on development of either miconazole (t = 0.23, p = 0.82) or levonorgestrel (t = −1.72, p = 0.087) were observed (Figure 1).

### Feeding inhibition

No effect on feeding rate was observed for levonorgestrel, whereas promethazine had a LOEC of 1.04 mg L^−1^ (Table 1). Feeding inhibition test on miconazole was not possible to conduct due to toxic effects on the algae used as a food source.

### RNA and DNA content

As expected, BL was a significant positive predictor of the individual RNA and DNA contents (Table 2), moreover both miconazole and promethazine had significant effects on RNA allocation. The RNA allocation per BL unit was significantly elevated in animals exposed to miconazole compared to the control, as indicated by the significant treatment × BL interaction (Table 2). In the promethazine treatment, the overall RNA content in the animals was elevated compared to the control, as indicated by significantly positive treatment effect (Table 2). No significant treatment effects were observed in animals exposed to levonorgestrel. No significant treatment effects on DNA content were observed for any of the substances (Table 2).

**Table 2:** General linear models testing treatment effects on the RNA – body length (BL) and DNA-BL relationships.

Pharmaceutical	Variable		RNA				DNA			
			Estimate	Df	F	p	Estimate	Df	F	p
Miconazole	Treatment		0.27	1	0.56	0.47	0.0089	1	1.88	0.19
	BL		0.92	1	35.27	<0.001***	0.13	1	9.00	0.0081**
	Treatment*BL		1.82	1	9.34	0.0075**	-	-	-	-
Promethazine	Treatment		0.12	1	4.62	0.047*	0.014	1	2.99	0.10
	BL		1.45	1	14.34	0.0016**	0.111	1	44.2	0.001***
Levonorgestrel	Treatment	0.019		1	1.62	0.22	0.010	1	1.83	0.19
	BL	1.33		1	18.34	<0.001***	0.12	1	5.86	0.027*

Tested concentrations were for miconazole 0.0023 mg L^−1^, for promethazine 0.059 mg L^−1^ and for levonorgestrel 1.02 mg L^−1^. Asterisks indicate significant level: p≤0.05 (*); p≤0.01 (**); p≤0.001 (***). All treatments were compared against the control.

### Gene expression

**Figure 2:**
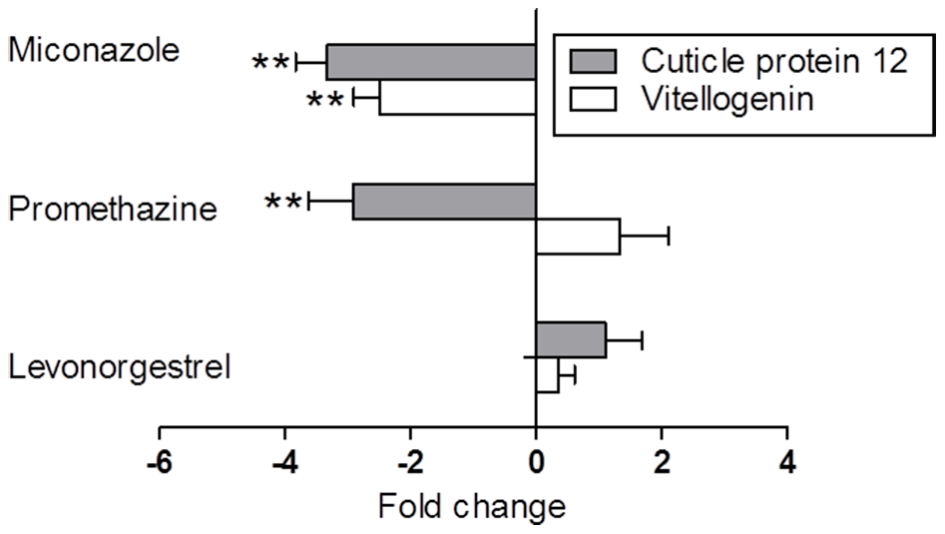
Gene expression changes. Change in gene expression of cuticle protein 12 and vitellogenin for *D. magna*, instar 3, exposed to miconazole (0.0023 mg L^−1^), promethazine (0.059 mg L^−1^) or levonorgestrel (1.02 mg L^−1^). The fold change (mean ± SD; *n* = 3) is shown in relation to the respective controls. Asterisks indicate significance level: p≤0.01 (**) determined by an unpaired t-test.

A three-fold decrease was observed for cuticle protein 12 expression in daphnids exposed to promethazine (t_4_ = 4.935, p<0.0078; Figure 2) and miconazole (t_4_ = 6.114, p<0.0036). Additionally, in the latter treatment, there was more than a two-fold decrease in vitellogenin expression (t_4_ = 6.763, p<0.0025), whereas this effect in promethazine was positive, although not quite significant (t_4_ = 2.511, p>0.066). By contrast, no significant changes were observed in the animals exposed to levonorgestrel (vitellogenin: t_4_ = 1.135, p>0.32; cuticle protein: t_4_ = 1.997, p>0.12; Figure 2).

### Chemical analysis

The results from the chemical quantification are presented in Table 3. Miconazole is a highly hydrophobic substance (log K_ow_ = 6.25); it was therefore not recovered from the aqueous phase. However, the dose-response relationships for mortality and reproduction indicate that the substance was present in the system and caused effects on the test animals.

**Table 3:** Measured concentrations (mg L^−1^) in the aqueous phase of the test medium before (Start) and after (End) exposures.

	Acute		Reproduction		RNA/Gene expression	
	Start	End	Start	End	Start	End
**Miconazole**	n.d. (9.3)		n.d. (0.022)	n.d. (0.022)	n.d. (0.0023)	n.d. (0.0023)
**Promethazine**	2.9 (3.1)		0.61 (0.53)	0.19 (0.53)	0.044 (0.059)	0.0053 (0.059)
**Levonorgestrel**					0.76 (1.02)	0.91 (1.02)

Water samples from acute and reproduction tests as well as for the incubations providing material for RNA/gene expression assays were analyzed. The nominal concentrations are presented in brackets. Measured concentrations for reproduction tests are mean concentrations from water samples taken at three time points during the 21-d exposure in connection to water change.

## Discussion

As hypothesized, the presence of drug target orthologs in *D. magna* was positively associated with the responses to the pharmaceuticals tested. In the animals exposed to miconazole and promethazine, which have identified drug targets in daphnids, significant effects on reproduction, mortality, RNA content and gene expression were observed. Moreover, promethazine had additional effect on developmental rate. By contrast, we found no effects of levonorgestrel, which lacks any identified drug target in this species. These findings indicate that drug target conservation is an important parameter for predicting toxic capacity and possible environmental effects of pharmaceuticals on non-target organisms.

For the standardized endpoints, we found miconazole to be the most toxic of the three pharmaceuticals tested with *D. magna*, with the lowest LC_50_ and LOEC values for mortality and reproduction, respectively. Even though the most toxic pharmaceutical could be identified by these tests, the effects at the individual level are as expected first observed when the molecular and biochemical effects are already severe [35].

Exposure to both miconazole and promethazine had positive effects on the RNA content in the exposed daphnids. This suggest an increased protein synthesis since individual RNA content is positively related to protein production capacity, which should ultimately lead to accelerated development and growth. Indeed, the number of ribosomes consisting largely of rRNA increases with increased protein synthesis rate [36]. Moreover, promethazine had a significantly positive effect on development, which agrees with the increase in RNA and probable increase in protein synthetic capacity. Although elevated RNA and protein synthesis levels may indicate increased growth rate and, hence, potentially higher fitness, they can also reflect increased synthesis of stress related proteins [37]. Heat shock proteins (hsps) belong to a family of evolutionary well-conserved proteins that are found in all living organisms [38]. Their function is to protect proteins and important cell structures from denaturation during stress situations. It is unlikely that the positive effects on RNA content were related to the stimulating effects on somatic growth and associated protein synthesis given the suppressed expression of cuticle protein and vitellogenin mRNA, i.e., mRNA coding for proteins involved in moulting and egg production processes, in the miconazole and promethazine treatments. One can speculate that the observed increase in RNA levels is a consequence of enhanced production of hsps induced by the pharmaceuticals. Moreover, exposure to miconazole resulted in increased RNA allocation per unit of mass as suggested by the increased slope of the relationship between RNA content and BL in the exposed daphnids. However, this increase was not related to any alterations in the allometric relationship between body mass and length as indicated by the homogenous slopes of the DNA-BL regressions (Table 2), which reflects an uniform increase of the cell number with BL. Rather, the observed increase in the slope of the RNA-BL regression following the miconazole exposure was a result of increased rRNA allocation to metabolic maintenance and synthesis of stress proteins that are not involved in routine metabolism. Even though protein synthesis is positively linked to RNA content, it also depends on the ribosomal activity, moreover, the relative importance of the ribosome number and activity may vary depending on the growth conditions [39]. Indeed, in fed rats, the rate of protein synthesis in liver tissue was entirely dependent on ribosomal activity, whereas in starved animals, ribosome number becomes an important controlling factor [39]. This indicates that under our experimental conditions, with food provided at relatively high levels (Table 1), the regulation of protein synthesis may largely rely on ribosomal activity and that the increase in RNA content observed in this study is not related to the increased synthesis of structural proteins needed for biomass build-up, but to the increased maintenance costs due to the stress.

Both miconazole and promethazine negatively affected the gene expression of the cuticle protein; moreover, miconazole also inhibited the vitellogenin expression. These two proteins are functionally related to reproduction and development [25]. Both miconazole and promethazine are known to target the calcium-binding CaM [17], [19], a highly conserved protein that in *D*. *pulex* has a predicted sequence similarity of 98% with the human CaM sequence [4]. CaM controls many physiological functions, among them ecdysteroid-production in crustaceans [40]. Hence, it is possible that the observed decrease in gene expression for cuticle protein is related to an inhibition of CaM activity. Also, the miconazole-induced suppression of vitellogenin expression may be related to the inhibition of CaM, similar to a decreased activity of CaM and concomitant effects on vitellogenin observed in frogs [41]. To establish whether CaM is involved in the observed responses, a controlled experiment with CaM gene expression and the protein activity measurements would be decisive. Further, the decreased vitellogenin expression is likely to be associated with the reduced reproductive potential as observed in *D. magna* exposed to fungicides propiconazole and feranimol [26], [42]. In these studies, decreased expression of genes associated with reproduction and development, among them vitellogenin and cuticle protein, was observed in the exposed animals. Our results support these findings and, together with the low effect concentration for reproduction, show that vitellogenin expression is a sensitive endpoint reflecting changes in reproduction.

In agreement with our hypothesis, no toxic effects for any of the endpoints investigated were observed at the tested concentrations for levonorgestrel, which lacks any identified drug target in *Daphnia*. Levonorgestrel has been found to cause adverse toxic effects on other vertebrates than humans, such as frogs and fish [20], [43] that have identified drug target orthologs [4] and binding receptors [3] for this drug. Estrogen receptor has not been found in *Daphnia spp.* [44] and Hannas et al. [45] found that vitellogenin gene expression in *D. pulex* was non-responsive to estrogenic activity of chemicals, which is in agreement with our findings.

By studying endpoints across different levels of biological organization, we observed complex responses to the pharmaceuticals tested. Acute tests rapidly provide information about the general toxicity; this, however, may be grossly underestimated [46], [47]. Reproduction tests provide more ecotoxicologically relevant information, as longer exposure time to lower concentrations is a more likely scenario in the environment. Biochemical and molecular responses may become manifested over shorter exposure times and effects can be observed at even lower concentrations. Even though the tested concentrations were much higher than commonly reported environmental concentrations (miconazole ∼8 ng L^−1^ [48], promethazine ∼2 ng L^−1^ [49] and levonorgestrel ∼7 ng L^−1^ [50]), this study demonstrates the principal feasibility to predict how potent a pharmaceutical may be based on the presence of a drug target ortholog.

As many other types of pollutants, pharmaceuticals may also act via narcosis [51]. The hydrophobic properties of miconazole [52] and promethazine [52], [53] (logP of 6.1 and 4.8, respectively; compared to levonorgestrel with logP: 3.8) as well as the observed toxic responses at relatively high concentrations indicate that narcosis could have been involved in some of the individual-level responses (immobility in particular) observed in this study. Ankley et al. [54] pointed out the importance of using endpoints and biomarkers associated with the MoA when assessing the toxicity of pharmaceuticals. However, for many pharmaceuticals the MoA in non-target species is not necessarily the same as in target species. Our results show that low-level endpoints, representing subcellular aberrations, such as RNA allocation and expression of genes related to egg production and cuticle building, can detect toxic effects at low concentrations when ortholog is present in the test species. Taken together, our results show that the increased toxic capacity of pharmaceuticals with conserved drug targets is detectable by endpoints of general toxicity.

Several authors [4], [14] have stressed the importance of incorporating the knowledge of drug target conservation in risk assessments of pharmaceuticals. Our results provide experimental evidence for the importance of this approach when applying endpoints of general toxicity and invertebrates as test organisms in risk assessments, although these species have fewer identified human orthologs than vertebrate species [4] and great uncertainties regarding their functionality. The drug target conservation approach could, and should, be strengthened by using adverse outcome pathway approach and molecular docking as essential tools to predict the affinity of pharmaceuticals to molecular targets and their toxic capacity towards non-target organisms [3]. Risk assessments of pharmaceuticals should focus on species that are at risk due to presence of drug target orthologs and thus facilitate selection of relevant test species for risk evaluation. This knowledge and increasing use of genomic tools would make risk assessments of pharmaceuticals more efficient.

## Supporting Information

Figure S1 **Immobilization of** ***D. magna*** **.** Mortality (%) for miconazole (A), promethazine (B) and levonorgestrel (C) after 48-h exposure. LC_50_ values are 0.3 mg L^−1^ (miconazole), 1.6 mg L^−1^ (promethazine) and >1.02 mg L^−1^ (levonorgestrel). Note different scales on the x-axis.
(TIF)

Figure S2 **Offspring production** ***D. magna*** **.** Reproduction effects of miconazole (A), promethazine (B) and levonorgestrel (C) measured as mean number of offspring per live parent after 21-d exposure. Error bars represents confidence interval (95%). Asterisk indicates significant difference in offspring production compared to control group (p≤0.05). Note different scales on the x-axis.
(TIF)

Table S1 **Measurements of pH and oxygen at the start and end of each exposure for acute tests, reproduction test and RNA/gene expression analysis.**
(DOCX)

Text S1 (DOCX)
