# Supplementary figures and images for: Are Pharmaceuticals with Evolutionary Conserved Molecular Drug Targets More Potent to Cause Toxic Effects in Non-Target Organisms?
Source: PLoS One. 2014 Aug 20;9(8):e105028. doi: 10.1371/journal.pone.0105028 (PMC4139295; doi:10.1371/journal.pone.0105028)

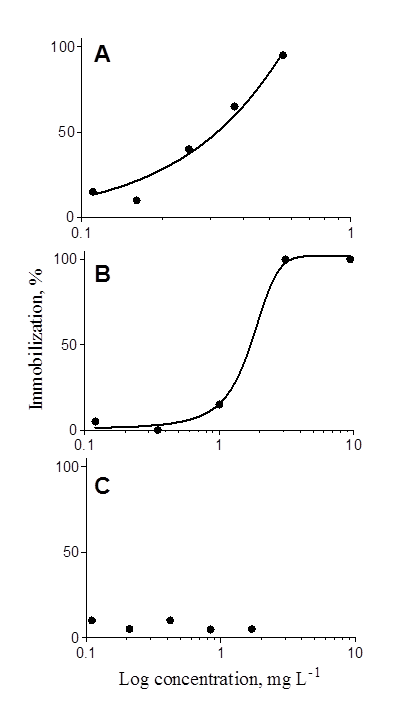

Supplement: Figure S1 — Immobilization of D. magna . Mortality (%) for miconazole (A), promethazine (B) and levonorgestrel (C) after 48-h exposure. LC50 values are 0.3 mg L−1 (miconazole), 1.6 mg L−1 (promethazine) and >1.02 mg L−1 (levonorgestrel). Note different scales on the x-axis. (TIF) [file pone.0105028.s001.tif]

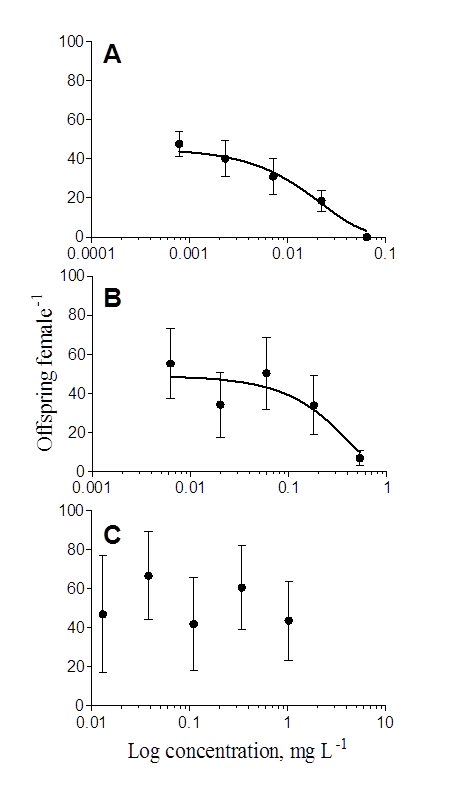

Supplement: Figure S2 — Offspring production D. magna . Reproduction effects of miconazole (A), promethazine (B) and levonorgestrel (C) measured as mean number of offspring per live parent after 21-d exposure. Error bars represents confidence interval (95%). Asterisk indicates significant difference in offspring production compared to control group (p≤0.05). Note different scales on the x-axis. (TIF) [file pone.0105028.s002.tif]
