# Supplementary material for: Are Pharmaceuticals with Evolutionary Conserved Molecular Drug Targets More Potent to Cause Toxic Effects in Non-Target Organisms?
Source: PLoS One. 2014 Aug 20;9(8):e105028. doi: 10.1371/journal.pone.0105028 (PMC4139295; doi:10.1371/journal.pone.0105028)
Supplement: Table S1 — Measurements of pH and oxygen at the start and end of each exposure for acute tests, reproduction test and RNA/gene expression analysis. (DOCX) [file pone.0105028.s003.docx]

**Table S1.** Measurements of pH and oxygen at the start and end of each exposure for acute tests, reproduction test and RNA/gene expression analysis.

|  | Acute (OECD 202) | | | | Reproduction  (OECD 211) | | | | RNA/Gene expression | | | |
| --- | --- | --- | --- | --- | --- | --- | --- | --- | --- | --- | --- | --- |
|  | *pH* | | *O_2_* (%) | | *pH* | | *O_2_* (%) | | *pH* | | *O_2_* (%) | |
|  | Start | End | Start | End | Start | End | Start | End | Start | End | Start | End |
| **Miconazole** |  |  |  |  |  |  |  |  |  |  |  |  |
| Control | 7.80 | 7.99 | 99 | 90 | 7.38 | 7.86 | 99 | 98 | 7.93 | 8.68 | 96 | 107 |
| Highest | 7.85 | 8.07 | 98 | 86 | 7.99 | 8.84 | 97 | 150 | 7.95 | 8.29 | 95 | 100 |
| **Promethazine** |  |  |  |  |  |  |  |  |  |  |  |  |
| Control | 7.63 | 7.81 | 99 | 91 | 7.67 | 7.80 | 93 | 98 | 8.12 | 8.13 | 111 | 115 |
| Highest | 7.71 | 7.70 | 89 | 94 | 7.81 | 7.62 | 103 | 101 | 8.12 | 8.31 | 114 | 113 |
| **Levonorgestrel** |  |  |  |  |  |  |  |  |  |  |  |  |
| Control | 7.63 | 7.81 | 99 | 91 | 7.56 | 7.76 | 98 | 102 | 8.05 | 8.05 | 116 | 99 |
| Highest | 7.64 | 7.78 | 96 | 96 | 7.84 | 7.88 | 95 | 101 | 7.88 | 8.21 | 117 | 102 |
