# Supplementary material for: Are Pharmaceuticals with Evolutionary Conserved Molecular Drug Targets More Potent to Cause Toxic Effects in Non-Target Organisms?
Source: PLoS One. 2014 Aug 20;9(8):e105028. doi: 10.1371/journal.pone.0105028 (PMC4139295; doi:10.1371/journal.pone.0105028)
Supplement: Text S1 — (DOCX) [file pone.0105028.s004.docx]

**Are pharmaceuticals with evolutionary conserved molecular drug targets more potent to cause toxic effects in non-target organisms?**

Sara Furuhagen, Anne Fuchs, Elin Lundström Belleza, Magnus Breitholtz, and Elena Gorokhova

*Department of Applied Environmental Science, Stockholm University, Stockholm, Sweden*

**Electronic supporting information**

1. **General information**

One of the main objectives within the Swedish research programme MistraPharma (www.mistrapharma.se) was to test a number of prioritized active pharmaceutical ingredients using a battery of standard ecotoxicological tests. The prioritization process is presented elsewhere [[1](#_ENREF_1)]. The three pharmaceuticals tested in this study fulfil the criteria presented by Fick et al.[[1](#_ENREF_1)].

1. **Determination and quantification of the pharmaceutical substances in aqueous media**

*Methods*

For the concentration determination of the pharmaceutical substances in the aqueous media, a liquid chromatography tandem mass spectrometry system (LC-MS/MS, TSQ Quantum Ultra Triple Stage Quadrupole, Thermo Scientific, USA) was used, equipped with electrospray (ESI) or atmospheric pressure chemical ionization (APCI)/atmospheric pressure photo ionization (APPI). Two LC-MS/MS methods were used due to the analytes ionization properties, ESI or APCI/APPI. Using ESI, 10 µL of diluted samples and calibration curve (10-1500 ng/mL, linearity above 0.99 for all substances) were injected to the analytical column (Hypersil Gold Aq, 50*2.1 mm, 5 µm, Thermo Scientific, USA) following a guard column (Hypersil Gold Aq, 10*2.1 mm, 5 µm). Using APCI/APPI, 10 µL of diluted samples and calibration curve (10-1500 ng/mL, linearity above 0.99 for all substances) were injected to the analytical column (Hypersil Gold Phenyl, 50*2.1 mm, 3 µm, Thermo Scientific, USA) following a guard column (Hypersil Gold phenyl, 10*2.1 mm, 5 µm). Mobile phase and flow gradients of the two methods are shown in Table A. Internal standard calibration (analyte and internal standard peak area ratios) was used to quantify the analytes and mass transitions; ion source information and quantification properties are shown in Table B.

| **Table A. LC mobile phase gradients and flows.** |
| --- |

| *ESI* | Time | A | B | C | µL/min |  |  |  |  |  |
| --- | --- | --- | --- | --- | --- | --- | --- | --- | --- | --- |
|  | 0.00 | 100 | 0 | 0 | 250 |  |  |  |  |  |
|  | 1.00 | 100 | 0 | 0 | 250 |  |  |  |  |  |
|  | 8.00 | 20 | 20 | 60 | 300 |  |  |  |  |  |
|  | 11.00 | 0 | 40 | 60 | 350 |  |  |  |  |  |
|  | 13.00 | 0 | 100 | 0 | 400 |  |  |  |  |  |
|  | 13.60 | 0 | 100 | 0 | 400 |  |  |  |  |  |
|  | 13.61 | 100 | 0 | 0 | 250 |  |  |  |  |  |
|  | 17.60 | 100 | 0 | 0 | 250 |  |  |  |  |  |
|  |  |  |  |  |  |  |  |  |  |  |
| *APCI/APPI* | Time | D | E | µL/min |  |  |  |  |  |  |
|  | 0.00 | 85 | 15 | 250 |  |  |  |  |  |  |
|  | 1.00 | 85 | 15 | 250 |  |  |  |  |  |  |
|  | 7.00 | 20 | 80 | 300 |  |  |  |  |  |  |
|  | 9.00 | 0 | 100 | 350 |  |  |  |  |  |  |
|  | 11.00 | 0 | 100 | 350 |  |  |  |  |  |  |
|  | 11.01 | 85 | 15 | 250 |  |  |  |  |  |  |
|  | 15.00 | 85 | 15 | 250 |  |  |  |  |  |  |
|  |  | | | | | | | | | |

| A: water, 0.1% formic acid (FA); B: acetonitrile, 0.1% FA; C: methanol, 0.1% FA; D: water; and E: methanol. |
| --- |

**Table B. LC-MS/MS and quantification properties for the substances tested.**

| Analyte | Precursor | Product  Q*^a^* | Product  q*^b^* | Ion source | IS*^c^* | LOQ*^d^*  ng/L |
| --- | --- | --- | --- | --- | --- | --- |
|  |  |  |  |  |  |  |
| **Miconazole** | 414.9 | 159.0 | 416.9🡪161 | ESI+ | Tramadol 13C;D3 | 5 |
| **Promethazine** | 285.1 | 86.3 | 198.0 | ESI+ | Promethazine D7 | 10 |
| **Levonorgestrel** | 313.1 | 109.2 | 91.3 | APCI/APPI+ | EE2 13C2 | 10 |
|  | | | | | | |

*^a^* Quantifier ion. *^b^* Qualifier ion. *^c^* Internal standards used in quantification. *^d^* Limits of quantification.

1. **References**

1. Fick J, Lindberg RH, Tysklind M, Larsson DGJ (2010) Predicted critical environmental concentrations for 500 pharmaceuticals. Regulatory Toxicology and Pharmacology 58: 516-523.

2. Barata C, Alanon P, Gutierrez-Alonso S, Riva MC, Fernandez C, et al. (2008) A Daphnia magna feeding bioassay as a cost effective and ecological relevant sublethal toxicity test for Environmental Risk Assessment of toxic effluents. Science of the Total Environment 405: 78-86.

3. Allen Y, Calow P, Baird DJ (1995) A mechanistic model of contaminant-induced feeding inhibition in Daphnia magna. Environmental Toxicology and Chemistry 14: 1625-1630.
